# Ozone: A Novel Sterilizer for Personal Protective Equipment

**DOI:** 10.7759/cureus.18228

**Published:** 2021-09-23

**Authors:** Lucas Stolle, Rohit Nalamasu, Robert Rodenbeck, Kyle Davidson, Caitlin Smarelli, Scot Rosko, Josh Wales, Jo Ann K LeQuang, Joseph Pergolizzi

**Affiliations:** 1 Molecular and Cellular Biochemistry, Oxford University, Oxford, GBR; 2 Physical Medicine and Rehabilitation, University of Nebraska Medical Center, Omaha, USA; 3 Research, Delta Faucet Company, Indianapolis, Indiana, USA; 4 Research, NEMA Research, Inc., Naples, USA; 5 Cardiology, Native Cardio Inc., Naples, USA

**Keywords:** covid, ppe sterilization, pandemic response, ozone, personal protective equipment

## Abstract

Objective: Personal protective equipment (PPE) is urgently sought during public health crises. It is necessary for the safety of both the patient and the healthcare professional. Yet during the recent COVID-19 pandemic, PPE scarcity in many countries, including the United States, has impacted the level of care for patients and the safety of healthcare personnel. Additionally, the implementation of mandatory mask mandates for the general public in many countries forced individuals to either reuse PPE, which can contribute to poor hygiene, or buy PPE in bulk and thereby contribute to the scarcity of PPE. In this study, we investigate the possibility of using a cost-effective ozone sterilization unit on contaminated N95 masks as an alternative to current sterilization methods.

Method: This protocol examined ozone’s ability to decontaminate N95 mask fabric that was exposed to a surrogate virus (*Escherichia coli bacteriophage MS2*). Once the sterilization unit achieves an ozone concentration of ~30 ppm, a 60-minute or 120-minute sterilization cycle commences. Following the sterilization cycle, we investigated the amount of viable virus on the slide using a viral plaque assay and compared it to a non-sterilized, control slide.

Furthermore, we carried out trials to investigate the safety of an ozone sterilization device, by measuring the levels of ozone exposure that individuals may experience when operating the sterilization unit post-cycle.

Results: We showed that a 120-minute sterilization cycle at ~30 ppm achieves a 3-log reduction in viral activity, thereby complying with industry and U.S. Food and Drug Administration (FDA) standards. Further, we demonstrated that when following our protocol, the ozone exposure levels for a simple sterilization unit to be used at home complied with federal and industry standards.

Conclusion: Ozone may have the potential to decontaminate masks and other PPE.

## Introduction

PPE (personal protective equipment) became a well-known acronym in the COVID-19 crisis as the global healthcare systems struggled to equip frontline clinicians with the equipment they needed; including gloves, masks, air-purifying respirators, goggles, face shields, gowns, and other items. Before the pandemic, PPE was widely available and considered disposable. As COVID-19 cases overwhelmed specific healthcare centers, PPE suddenly became both scarce and urgently needed in large quantities [1].

As PPE may not be reused without sterilization, which could result in the loss of infection prevention, large quantities of PPE are needed to manage large-scale infections. Due to the size of this current pandemic, millions of face masks, gloves, and gowns are required [2]. But even prior to the COVID-19 pandemic, Vozzola et al. proposed that reusable isolation gowns could reduce energy consumption by 28%, greenhouse gas emissions by 30%, and the production of solid waste by 93% compared to disposable isolation gowns [3]. This particular proposal was driven by concerns about preserving the environment, rather than managing shortfalls of isolation gowns. Reusable isolation gowns are available and the decision for a healthcare facility to use them is largely based on cost considerations, logistics, and preferences.

Single-use N95 respirator masks were in such short supply early in the pandemic that ways to decontaminate them for reuse were considered. Investigators examined the use of ultraviolet (UV) radiation (260 to 285 nm), heat (70°C), 70% ethanol, and vaporized hydrogen peroxide (VHP). Effects of these methods on the inactivation rate of the SARS-CoV-2 virus on the filter fabric and stainless steel of an N95 face mask were measured, in addition to a quantitative fit-test evaluation of the respirator [4]. Both VHP and ethanol resulted in rapid inactivation of the virus on the fabric and stainless steel; UV worked to inactivate the virus quickly from stainless steel, but more slowly from fabric. On the other hand, heat had the opposite effect, with the virus becoming inactivated more rapidly on N95 cloth than steel. Fit-test parameters showed good results after a single round of decontamination, but the second-round results showed diminished performance for ethanol- and heat-decontaminated N95 masks. The UV-treated and VHP-treated N95 masks did well in fit tests for two rounds of decontamination and provided acceptable performance after the third round. It was reported that the N95 masks could be decontaminated and reused up to two times for dry heat and up to three times for UV-light and VHP-treatments [4].

Vaporized hydrogen peroxide is an industry-standard for decontamination in laboratories, research, pharmaceutical organizations, and medical facilities because of its low toxicity and the ease of catalytic reaction between water and oxygen [2]. Project BREATHE (Better Respiratory Equipment using Advanced Technologies for Healthcare Employees) was initiated in 2009 to determine if N95 respirators and other types of filtering face-piece respirators (FFRs) could be safely and effectively decontaminated for reuse [2]. This study was done prior to the COVID-19 pandemic in preparation for possible emergencies. Prolonged exposure to VHP over multiple cycles did not degrade the filter in the mask but did degrade elastic within the mask straps [2].

With these successful results, governmental authorities began to employ VHP in the decontamination of N95 masks. Subsequently, the US FDA issued several emergency use authorizations (EUAs) for VHP-based N95 mask decontamination units [5,6]. At the time of writing, all N95 sterilizers covered under the FDA’s EUA are VHP based. These predominantly differ in the VHP concentration, phase, or in additives that are combined with the VHP [6].

Our study assessed the use of ozone to sterilize PPE as a cost-effective alternative to the VHP-based methods. As ozone does not humidify the masks (unlike any vapor-based protocol) and requires no intense heat, it may offer a gentler decontamination of N95 masks [7,8]. Thus, we hypothesized that ozone could be employed for more decontamination cycles, without diminishing the fit factor or filtration capacity of the mask.

Ozone is a widely accepted inactivator of bacteria and, in particular, viruses, which are the most vulnerable to ozone prior to cell association. Ozone can effectively incapacitate the virus and prevent infection of the host cell [9]. In water or a humid environment, ozone's decomposition into hydroxyl, superoxide, and hydroperoxyl radicals contributes to its high oxidizing power [10], which is thought to be capable of lysing viral coat proteins and even lead to the complete destructive inactivation of the virus. Secondarily, ribonucleic acid (RNA) shearing may also be observed, though this is thought to be a remnant of the aggressive free radical lysis [11].

More recent papers suggest that ozone may disrupt the redox state of cysteine residues within the fusion or attachment proteins around the virus. Rowen et al. note that coronaviruses, SARS Cov-2 included, have an abundance of cysteine residues in their spike proteins that require a reduced (R-SH), as opposed to an oxidized (R-S-S-R) cysteine residue for successful infection [12]. Thus, ozone may prove a valuable method for the sterilization of PPE.

The viricidal activity of ozone has also been positively correlated to the relative ambient humidity, with more humid environments - or complete immersion in water - presumably increasing the formation of hydroxyl radicals and thus viricidal activity [10]. In this study, we wish to examine whether ozone at low ambient humidity is similarly capable of inducing a significant reduction in viral activity, for a more gentle and less expensive alternative to currently employed VHP treatments.

## Materials and methods

Test microorganism

The test microorganisms were obtained from ATCC® (American Type Culture Collection) and included Escherichia coli bacteriophage MS2 (ATCC® 15597-B1™) as a viral surrogate and Escherichia coli strain C-3000 (ATCC® 15597™), as a permissive host cell system. MS2 Bacteriophage is a non-enveloped positive-stranded RNA virus of the bacteriophage family Leviviridae. Bacterial cells including E. coli (strain C-3000) serve as a host for MS2 bacteriophage. Its small size, icosahedral structure, and environmental resistance have made MS2 ideal for use as a surrogate virus (particularly in place of picornaviruses such as poliovirus and human norovirus) in water quality and disinfectant studies [13-16]. The respective incubation and phage propagation procedures were obtained from the supporting documentation for each product, and all incubation media were tested for and passed standard sterility tests. Care was taken in the selection of materials for the enclosure and shelf carriers, such that the ozone load (ozone consumed by the materials), did not adversely affect the achievement of desired ozone levels.

Sterilization unit

**Figure 1:**
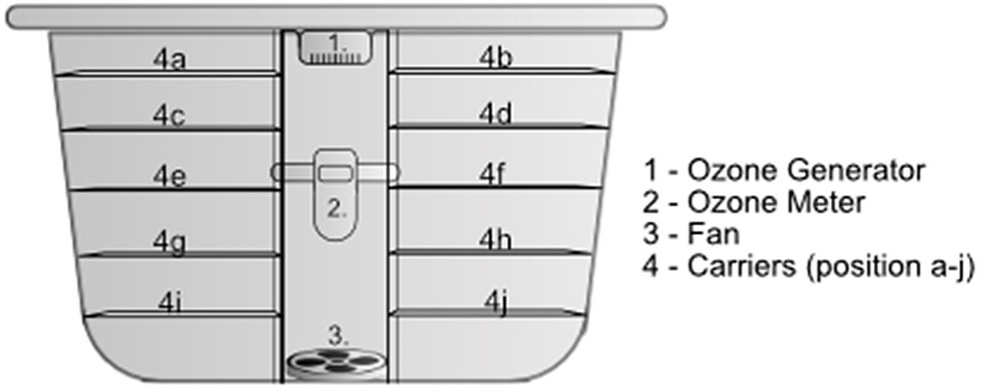
Sterilization unit box assembly

The sterilization unit was provided by the study sponsor (Delta Faucet Company, Indianapolis, Indiana, USA) and included an ozone sterilization box, an ozone generator (Whitetail’R® ScentPURGE™ 50, Matheson' Brand LLC, Plymouth, WI, USA), a shelf carrier setup, and a fan for even ozone distribution within the unit. The assembly of the unit is shown in Figure 1. 

The N95 masks were cut into 4 cm x 4 cm pieces, autoclaved for sterilization purposes, and allowed to dry completely prior to testing. The viral stock was diluted in phosphate-buffered saline (PBS) to target an inoculum concentration of ~1x107 plaque-forming unit (PFU). A 0.5 mL of the inoculum was applied via micropipette to the surface of the N95 slides. The inoculated carriers were then allowed to dry under ambient conditions within a biological safety cabinet for 40 minutes prior to their use. Only fully dried slides were used for subsequent experimentation. Three test slides were applied per sterilization cycle, with ozone exposure times of 60 minutes or 120 minutes. To test for sterilization discrepancies within the sterilization unit, different rack heights and positions were tested. For most cycles, racks 4a, 4e, and 4j (as seen in Figure 1) were used, with the slides on 4a and 4e being juxtaposed (i.e., one placed at the front and the other at the rear of the respective rack). The rack heights in initial testing resulted in a consistent difference in viricidal activity between lower and high rack heights (with the top rack showing less sterilization potential), so a fan was incorporated into the system. The use of the fan was important, and after it was added the sterilization was approximately equal on both rack heights. In these studies, a fan (NMB Technologies 3115PS-12W-B10-A00, Novi, MI, USA) at a flow rate of 19.4 cubic feet per minute (CFM) was used. The fan had a simple on/off control and no means to regulate speed. Since the preset speed produced good results, the team did not investigate the issue of fan speeds further.

During each cycle, the ozone concentrations were measured by an ozone sensor (Aeroqual™ S-500, Auckland, NZ) in five-minute intervals. Each cycle started with 30 minutes of build-up time, allowing the sterilization unit to reach its maximum ozone concentration of ~32 ppm, which was achieved by 35 minutes. This concentration was then maintained for either 60 or 120 minutes.

Control 4 cm x 4 cm N95 mask pieces were prepared alongside the test slides but were kept in an incubator for the duration of the sterilization cycle. These control slides were then used to compare the viral-load reduction relative to those undergoing a sterilization cycle. The structural integrity or "fit" of the mask following sterilization was not tested, which warrants further study as it is crucial for PPE integrity.

Viral quantification

After the contact time, carriers were harvested in 20 ml of PBS supplemented with 0.1% surfactant (Triton X-100). The 50 ml conical tubes, containing the harvested carrier and liquid media were vortexed for 30 seconds to recover the test microorganism from the carrier and suspend the MS2 into the liquid media. Subsequently, phage concentrations were quantified using a standard viral plaque assay, with Escherichia coli strain C-3000 (ATCC® 15597™) used as the permissive host cell system. The PFU per milliliter (PFU/ml, see Eq. 1) and PFU per carrier (PFU/carrier, see Eq. 2-3) were determined using the average plate count (APC) and the applicable dilution factors (DF). The percentage reduction (see Eq. 4) and the log reduction (see Eq. 5) on the other hand were determined from the average number of viable test microorganisms on the control carriers immediately after inoculation and after the contact time (denoted as B) and the number of viable test microorganisms on the test carriers after the contact time (denoted as A).

Calculations

**Figure 2:**
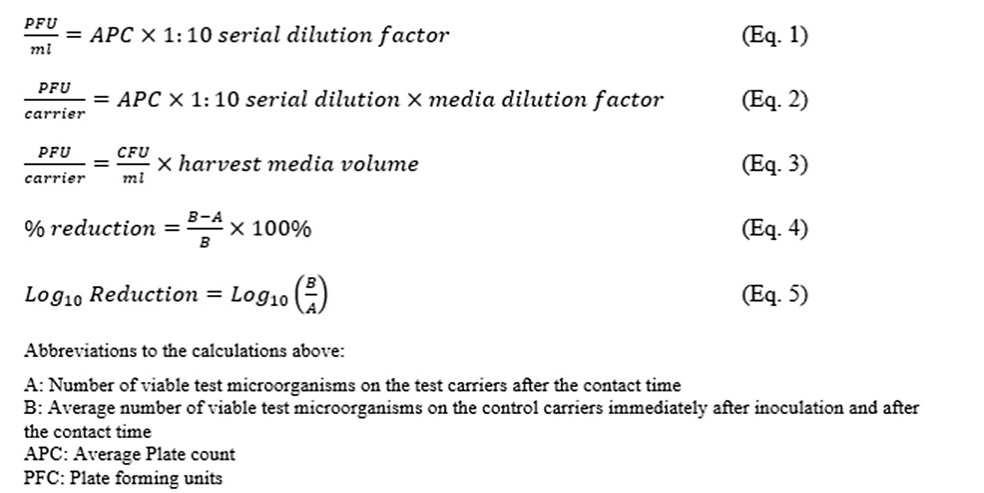
Calculations Eq: Equation

The following equations (Eq) in Figure 2 were used for calculations described in these studies. 

Health and safety considerations

Since the use of ozone-based sterilization of PPE may be suitable not only for healthcare professionals but also for home use, it was important to consider the levels of ozone exposure during normal and incorrect use of the unit. Predominant safety considerations involved the quantity and duration of ozone exposure after opening the sterilization unit post-cycle (i.e., with an internal concentration of ~30-32 ppm), as well as the effects of leaving the lid half or fully open during the sterilization cycle.

Exposure levels for each scenario were explored using an ozone sensor (Aeroqual TM S-500) placed 3 ft away from the sterilization unit on the floor, 3 ft away from the sterilization unit, and elevated at a height of 1.3 ft (to simulate a pet or a toddler) or 1 ft directly above the sterilization unit (to mimic someone bending over and opening the container).

## Results

N95 slide sterility for a 60-minute cycle

The 60-minute sterilization cycle produced a significant difference in the number of viable test microorganisms on the slide between the sterilized and the unsterilized carriers. The average percentage reduction (see Eq. 4) between the 60-minute-sterilized and the Time Zero control was 99.28%, with a Log10 reduction of 2.15.

N95 slide sterility for a 120-minute cycle

In contrast, the 120-minute sterilization cycle produced a greater difference between sterilized and unsterilized N95 slides. A 99.904% viral reduction was reported between the sterilized and the control carrier, with a significant Log10 reduction of 3.02.

Health and safety tests

At low run-times, where the ozone concentration failed to exceed 12 ppm, the ozone has been found to disperse slowly when the unit is opened, with maximum ozone levels not exceeding 0.060 ppm.

When used as suggested in our sterilization process (with concentrations of ~30ppm), the exposure levels immediately following the opening of the unit (with the sensor placed 1 ft above the unit to mimic a person bending over and emptying the container after the sterilization cycle) briefly reached 0.054 ppm define acronym. On the other hand, when the same experiment is performed for longer durations, or with multiple ozone generators (up to six), the ozone exposure levels immediately above the unit can exceed 0.600 ppm.

## Discussion

The viral strain employed in the present study - Escherichia coli bacteriophage MS2 (ATCC® 15597-B1™) - was a surrogate virus for common viral strains including norovirus. In this proof-of-concept study, we demonstrated that extended exposure of PPE sample slides to ozone at 32 ppm, is capable of efficiently reducing the viral load on the PPE. FDA standards mandate that for a putative sterilization technique to be deemed appropriate, the viricidal activity has to demonstrate a log reduction [6]. In these studies, a 12-minute ozone exposure to PPE samples led to a 99.904% and 3.02 log reduction in viral activity, thus meeting or exceeding the FDA standards.

Additionally, when following our proposed protocol, the ozone exposure levels comply with the maximum recommended exposure levels of <0.100 ppm authorized by the United States National Institute for Occupational Safety and Health (NIOSH) and the United States Occupational Safety and Health Administration (OSHA) [17]. Nevertheless, the use of ozone should be restricted to well-ventilated rooms, and direct or close contact with the unit should be minimized to limit ozone exposure as much as possible.

Our study was primarily directed at discovering the potential of ozone in the disinfection of PPE. The promising data from this small-scale investigation on a SARS-CoV2 surrogate virus - MS2 bacteriophage [16] - suggests further studies that may compare ozone to currently employed decontaminants, such as VHP.

This is a proof-of-concept study, and there are several limitations that must be noted. Primarily, the inaccessibility of SARS-CoV-2 required the use of a surrogate virus for our testing. Additionally, with only three repetitions, this study cannot make any broad statistically supported claims. Finally, a common issue in PPE sterilization and reuse is the loss of structural integrity and “fit” of the mask, as well as the disintegration of certain susceptible components [4]. Assessing the effect of ozone treatment on the fit of masks will be crucial in subsequent studies to assure that this treatment not only sterilizes but also maintains the structural integrity of the PPE. 

The ozone decontamination device described here was set up as an open-loop system and cost <$200 to produce and a similar but closed-loop system would cost approximately $600. Biocontamination systems available online may cost 10 times that or more. 

## Conclusions

The COVID-19 pandemic has caused disruption in almost all aspects of our daily life and forced us to change the way we approach new problems. Among these issues was the disruption of supply chains and the limited supply of PPE for both healthcare personnel and the general public. While other methods for sterilization of PPE have been proven, our results confirm the potential of the cost-effective ozone system as a putative sterilizer for PPE. Ozone at 32 ppm in a simple sterilization unit was tested against MS2 Bacteriophage for two distinct sterilization-cycle lengths (60 and 120 minutes). In these tests, the 120-minute sterilization cycle reduced viral load by 99.904% (Log10 reduction of 3.02), which is sufficient to comply with FDA guidelines for a Tier 3 N95-respirator decontamination system. Therefore, ozone may offer a cost-effective way to sterilize N95-respirators and potentially other PPE both in healthcare systems and for home use. Nonetheless, further study is warranted to assess the effect of ozone on longevity and fit of PPE, as well as assessing the optimal implementation of such systems in healthcare and laymen environments.
